# Effect of different salinity on seed germination, growth parameters and biochemical contents of pumpkin (*Cucurbita pepo L*.) seeds cultivars

**DOI:** 10.1038/s41598-024-55325-w

**Published:** 2024-03-22

**Authors:** Hasan Ali Irik, Gülsah Bikmaz

**Affiliations:** 1 Department of Biosystems Engineering, Faculty of Agriculture, University of Erciyes, Kayseri, Turkey; 2 Department of Biosystems Engineering, Institute of Graduate School of Natural and Applied Sciences, University of Erciyes, Kayseri, Turkey

**Keywords:** Pumpkin seed, Salinity, Germination, Seedling vigour index, Prolin, DPPH, Physiology, Plant sciences, Psychology

## Abstract

Soil and water salinity is an important limiting factor affecting yield and production levels in arid and semi-arid areas. Salt tolerance during germination is an important parameter that also affects the other plant development stages. In this respect, this study was designed to determine the responses of pumpkin seed varieties (Develi, Ürgüp, Hybrid) to different NaCl salinities. The study was carried out in 2022 in the laboratory of Biosystems Engineering Department of Erciyes University in randomized plots design with 3 replications. Experiments were conducted with 5 different water salinity. Germination percentage (GP), germination index (GI), mean germination time (MGT), seedling vigor index (SVI), ion leakage (Il), radicula length (RL) and plumule length (PL), root and shoot fresh and dry weights and some mineral composition (Na, K, Ca) were examined. Proline, antioxidant capacity, total phenolic and DPPH content were significantly affected by salinity. In scatter plot correlation analysis SVI a positive correlation was observed between GP (r^2^ = 0.774), GI (r^2^ = 0.745), RL (r^2^ = 0.929), FRW (r^2^ = 0.837), FSW (r^2^ = 0.836), DRW (r^2^ = 0.894), AC (r^2^ = 0.747), TP (r^2^ = 0.640) and DPPH (r^2^ = 0.635). It was determined that there were negative correlations between SVI and MGT (r^2^ = − 0.902), II (r^2^ = − 0.588), DSW (r^2^ = − 0.682) and PR (r^2^ = − 0.344). Present findings revealed that investigated parameters were significantly affected by increasing salinity levels. While Hybrid cultivar was the most affected by salinity, Develi cultivar was found to be resistant to saline conditions.

## Introduction

Salt stress is an important abiotic stress factor that limits crop productivity through negative impacts on plant growth and development especially in arid and semi-arid regions. It was reported that approximately 19.5% of irrigated lands and 2.1% of dry lands were affected by salt stress. In addition, saline lands are continuously increasing mainly due to improper irrigation management practices^1–3^. Salinity-induced osmotic and ion stress negatively influence plant growth and development and such negative impacts largely depend on type of salt, level and duration of salt stress, genotype and developmental stage of the plant exposed to salt stress^4^. Salinity alters various metabolic processes and especially photosynthetic activity of the plants, then reduce the chance of survival. While some plants are sensitive to saline conditions, some survive by tolerance mechanisms induced by various physiological, biochemical and molecular responses. Plants provide tolerance mechanisms to salinity as physiological and biochemical responses. Selective accumulation or excretion of ions, control of ion uptake in roots and transmission to shoots, and accumulation of these ions in certain parts of the plant and cells. Additionally, antioxidant systems are activated with the synthesis of osmotic regulators. These molecular responses provide activation or inactivation of various genes via signal transduction pathways. Resultant physiological, biochemical and molecular responses provide the maintenance of salt regulation in plants^5^.

Seed germination and seedling growth stages are the most important and most vulnerable stages in the life cycle of plants. Therefore, salinity studies have focused on these two main stages and these stages are taken into account when determining the salt resistance of plants^6^. Previous studies have also reported that salinity stress has a negative effect on germination and growth parameters in plants (Ref.^7^ in lettuce; Ref.^8^ in squash; Ref.^9^ in pepper). Increasing salinity concentration reduces the osmotic potential, limiting germination percentage, germination rate and root development. It also causes ion toxicity and oxidative stress^10,11^. Plants can combat oxidative stress through enzymatic (such as catalase, ascorbate peroxidase, and superoxide dismutase) and non-enzymatic (such as carotenoids, proline, α-tocopherol, and ascorbic acid) antioxidants^12,13^.

Majority of pumpkin species of *Cucurbitaceae* family can be grown without any problems in Turkey. Some pumpkin species are consumed fresh, while others are consumed as snacks. Majority of seed pumpkins grown in Turkey belongs to *Cucurbita pepo* L. species and a small number of them belongs to *Cucurbita moschata* species^14^. Seed pumpkin production has a significant place in income sources of Central Anatolian farmers. Of about 57,184 tons of seed pumpkin production of Turkey in 2020, 16,920 tons were produced in Kayseri province^15^. Such a number corresponds to 29.65 of country production. Pumpkin seeds are mostly consumed as appetizer. However, with a rich composition, they are also used in cosmetics, pharmaceutical, health and food industries^16^. Just because of insufficient or deficit water resources, seed pumpkin cultivation is practiced under dry (rain-fed) conditions in arid and semi-arid regions. Seed pumpkin cultivation is increasing its attractiveness in these regions day by day due to its profitability as compared to cereals, its ease of storage and marketing. This study was conducted to determine the germination response of pumpkin seeds against salinity.

## Material and method

This study was carried out in the laboratories of Biosystems Engineering Department of Erciyes University in February 2022. Pumpkin seeds to be used were obtained from local farmers. Develi, Ürgüp and Hybrid (Ukrainian type) cultivars, commonly used genotypes of the region, were used as the primary materials of the study. The study was conducted in accordance with the guidelines specified by the International Seed Testing Association (ISTA).

Saline waters were obtained with the use of NaCl salt. Five different salt concentrations (S_1_, 0.3 dS/m (control); S_2_, 2.5 dS/m; S_3_, 5 dS/m; S_4_, 7.5 dS/m and S_5_, 10 dS/m) were prepared.

Before the initiation of germination tests, pumpkin seeds were sterilized with 10% sodium hypochlorite for 10 min and sterilized seeds were passed through distilled water 5 times for disinfection. Disinfected seeds were placed on 20 × 20 cm filter papers with 25 seeds on each. Experiments were conducted in randomized plots design with 3 replications. Solutions of 20 ml were added to each treatment and germination papers were placed in ziplock bags to prevent evaporation. Germination was carried out in a completely dark incubator at 20 °C. Seeds were counted at the same time each day and seeds with a rootlet length of 2 mm were considered as germinated. To prevent dry out of filter papers, 10 ml of solution was added to each treatment every other day. The total germinated seeds were counted on the eighth day^17^.

The following parameters were studied:

Seedling shoot and root length of ten randomly selected seedlings from each replication were measured at the time of harvest. Shoot dry weight and root dry weight were recorded after drying at 65 °C for 72 h.

Germination percentage (GP),

1 $${\text{GP}}= \frac{Number \,\,of \,\,normally \,\,germinated \,\,seeds}{Total \,\,number \,\,of \,\,seeds}\times 100$$

Germination index (GI),

2 $${\text{GI}}=\sum \left({G}_{t}/{T}_{t}\right)$$

where G*t* is the number of seeds germinated on day *t*, T*t* is the number of days.

Mean germination time (MGT),

3 $$\mathrm{MGT }=\sum \left(Ti\times Ni\right)/ \sum Ni$$

where Ni is the number of newly germinated seeds at time Ti.

Seedling vigor index (SVI),

4 $$\mathrm{SVI }= Mean \,\,germination\,\, percentage\times mean \,\,seedling\,\, lenght,$$

### Mineral composition

The K, Ca, Na contents of the plant samples were analyzed with nitric acid-hydrogen peroxide (2:3) acid in 3 different steps (1st step; 5 min at 75% microwave power at 145 ºC, 2nd step; 90% microwave power at 180 ºC). 10 min and the 3rd step (10 min at 100 ºC at 40% microwave power) after being exposed to a 40 bar pressure resistant microwave wet combustion unit (Anton paar microvawe)^18^ (P, K, Ca, Mg, Na, Fe, Mn, Zn, Cu and B) were determined by reading on the ICP OES spectrophotometer (Inductively Couple Plasma spectrophotometer) (Agilent,5110 Optima, ICP/OES)^19^.

### Prolin content

In order to determine the amount of Proline in pumpkin seed^20^, by applying partial modifications to the method. 0.1 g of dried plant tissue was crushed with a 3% solution of 5 ml of sulfosalicylic acid using a mortar. The extract was centrifuged at 15000×*g* for 10 min. 2 ml of supernatant was added to each tube in duplicates and then 2 ml of glacial acetic acid and 2 ml of acetic acid, phosphoric acid, ninhydrin solution were added to the tubes and mixed. The tubes were boiled for 1 h, at the end of this period, instant cooling was done and 4 ml of toluene was added to the samples and mixed. The toluene portion was taken into a glass cuvette and read at 520 nm.

### Antioxidant capacity

Reference^21^ by applying partial modifications to the method. In this context, the reagent solution was first prepared. Combine 0.6 M sulfuric acid (30 ml), 28 mM sodium phosphate (28 ml) and 4 mM ammonium molybdate (40 ml) and make up to 100 ml with water. The solution must be prepared fresh. Afterwards, 0.4 ml of sample was mixed with 4 ml of reagent solution and after vortexing the test tubes, they were incubated in a water bath at 95 °C for 90 min. After rapid cooling in cold water, the absorbance values of the samples were measured at 695 nm with a UV–vis spectrophotometer.

### Total phenolic content

Determination of the total phenolic content of the samples was carried out by applying partial modifications to the method proposed by Ref.^21^. In this context, 0.2 ml of the liquid extract was taken, 1.8 ml of distilled water and 1 ml of diluted (1:10) Folin Ciocalteu reagent were added to it. After 5 min, 2 ml of 2% Na_2_CO_3_ was added to the samples and after the tubes were tightly closed and vortexed, they were left to incubate in the dark for 2 h. At the end of the incubation, the absorbance values of the samples were read with a spectrophotometer (UV-1700, Shimadzu, Japan).

### DPPH radical scavenging activity

The antiradical activity of the samples was carried out by applying partial modifications to the method proposed by Ref.^22^. For this purpose, 0.1 ml of the samples were added to the test tubes and mixed with 3.9 ml of DPPH (Sigma, USA) solution (prepared in 0.1 mM and methanol), then covered with aluminum foil and left in a dark environment for 30 min. At the end of the period, the absorbance values of the test tubes were determined at 517 nm in the UV–Vis spectrophotometer zeroed with ethanol.

$$\% Inhibition=\left(\frac{Ac-As}{Ac}\right)\times 100$$

where* A*_*c*_ is the control absorbance nad* A*_*s*_ is the sample absorbance. Radical scavenging activity values were given as mg AAE/kg using the ascorbic acid calibration curve.

Experimental data were subjected to analysis of variance with the use of Jump 17 pro statistical software. Significant means were compared with the use of Duncan’s test. In addition to, principal component analysis and correlation analysis were performed.

## Results and discussion

### Effect of salinity on germination parameters

Germination percentage (GP) was statistically affected by both salinity and cultivar. Salinity × cultivar interaction did not have any significant effects on GP. It was observed that GP was affected by salinity for all seed pumpkin cultivars (Table 1). With increasing salinity levels, GP decreased in all cultivars. Among the cultivars, the highest GP (88%) was obtained from Develi cultivar and the lowest (66%) from Hybrid cultivar. In Ürgüp cultivar, GP was 71%. For salt doses, the highest GP (81%) was seen in S_1_ and lowest (68%) in S_5_ treatments. In terms of interactions, the highest GP (96%) was obtained from S_1_ of Develi cultivar and the lowest (61%) from S_5_ of Hybrid cultivar. Reference^23^ reported that GP was severely affected with increasing salinity levels in paddy. It was reported in previous studies that GP values decreased with increasing salinity levels in lettuce cultivars^24^, Tunisian squash^8^ and watermelon cultivars^6^.

**Table 1:** Effect of salinity on some germination parameters.

Cultivars	Treatments	GP (%)	GI	MGT (day)	SVI	Ion leakage (%)
Develi	S_1_	96^a^	29.5^a^	2.98^d^	1425.7^a^	43.40f.
	S_2_	93^ab^	28.0^ab^	3.24^bcd^	1174.7^ab^	50.31^ef^
	S_3_	88^ab^	27.1^ab^	3.36^abcd^	903.7^bcd^	54.18^e^
	S_4_	84^abc^	23.6^abc^	3.70^abcd^	790.7^bcd^	56.38^e^
	S_5_	80^abc^	21.9^abcd^	3.72^abcd^	620.0^d^	65.37^d^
Ürgüp	S_1_	75^abc^	19.3^bcd^	3.13^ cd^	1099.6^abc^	19.76^ h^
	S_2_	73^abc^	21.0^abcd^	3.24^bcd^	1048.6^abc^	20.86^ h^
	S_3_	73^abc^	20.2^bcd^	3.46^abcd^	883.3^bcd^	22.31^ h^
	S_4_	69^bc^	20.7^abcd^	3.43^abcd^	797.4^bcd^	25.60^gh^
	S_5_	63^c^	20.1^bcd^	3.47^abcd^	656.8^d^	32.57^ g^
Hybrid	S_1_	73^abc^	17.6^ cd^	3.39^abcd^	865.0^bcd^	65.72^d^
	S_2_	71^bc^	19.2^bcd^	3.66^abcd^	728.8^ cd^	71.49^ cd^
	S_3_	63^c^	14.0^d^	4.12^a^	588.5^d^	77.99^bc^
	S_4_	63^c^	14.8^ cd^	3.94^abc^	559.3^d^	86.19^b^
	S_5_	61^c^	16.2^ cd^	4.05^ab^	522.5^d^	96.99^a^
General means	Cultivars					
	Develi	88a	26.03a	3.40b	982.97a	53.93b
	Ürgüp	71b	20.26b	3.35b	897.17b	24.22c
	Hybrid	66b	16.35c	3.83a	652.82c	79.68a
	Salinity					
	S_1_	81a	22.10	3.17c	1130.12a	42.96e
	S_2_	79ab	22.76	3.38bc	984.05a	47.55d
	S_3_	75abc	20.42	3.65ab	791.86b	51.50c
	S_4_	72bc	19.70	3.69a	715.80bc	56.06b
	S_5_	68c	19.40	3.75a	599.78c	64.97a
Significance						
Salinity (S)		**	ns	**	**	**
Cultivar (C)		**	**	**	**	**
S × C		ns	ns	ns	ns	**

*p < 0.05, **p < 0.01, *ns* not significant.

While the germination index (GI) values were not affected by salinity and salinity × cultivar interaction, the effect of cultivars was found to be significant (p < 0.01) (Table 1). GI, an indicator of resistance, varied significantly with the cultivars. In terms of cultivars, GI value was found to be 26.03 in Develi cultivar, 20.26 in Ürgüp cultivar and 16.35 in Hybrid cultivar. Present data revealed that Develi cultivar had a higher salt resistance than the others.

While salinity and cultivar had significant effects on mean germination time (MGT) (p < 0.01), salinity × cultivar interaction did not have any significant effects on MGT. It was observed that MGT increased with increasing salt doses in all cultivars (Table 1). MGT values were found as 3.40, 3.35 and 3.83 in Develi, Ürgüp and Hybrid cultivars, respectively. There was no significant difference between Develi and Ürgüp cultivars. In all varieties, the lowest MGT value was obtained from S_1_ treatments, while the highest values were obtained from S_5_ treatments. Present findings on MGT comply with the results of previous studies^25,26^.

Seedling vigor is a complex agronomic trait with various indicators such as germination rate, final germination percentage and germination index during the seed germination stage, root length during early seedling growth, shoot length, fresh weight and dry weight^26^. Seedling vigor index (SVI) is defined as the seed characteristics that designate the rapid and uniform emergence and development potential of normal seedlings^27^. Both salinity and cultivars had significant effects on SVI (p < 0.01), while salinity × cultivar interaction did not have any significant effects on SVI (Table 1). SVI decreased in all cultivars with increasing salt doses. Especially after 2.5 EC salinity, serious decreases were observed. SVI value was calculated as 982.97 for Develi cultivar, 897.17 for Ürgüp cultivar and 652.82 for Hybrid cultivar. In terms of salt doses, the highest value (1130.12) was seen in control treatment and the lowest (599.78) in 10 EC treatment. SVI values varied between 1425.7 and 620 in Develi cultivar, between 1099.6 and 656.8 in Ürgüp cultivar and between 865 and 522.5 in Hybrid cultivar. Reference^9^ in their study on peppers, they obtained the highest SVI value from the control (0 nM) treatment and the lowest SVI value from the treatment with 200 mM NaCl salinity. In another study conducted on medicinal pumpkin, it was reported that the SVI value decreased as salinity stress increased^28^.

Ion leakage is an indicator of stability and integrity of cell membrane and is used as an important parameter that reveals stress tolerance of plants^29^. Ion leakage is determined to reveal the relationship of membrane integrity with environmental stresses, growth, development and genotypic changes. Stress-induced leakage allows the detection of tissue damage^30^. Cultivar, salinity and salinity × cultivar interaction generated significant differences in ion leakage values (p < 0.01) (Table 1). In terms of cultivars, the highest ion leakage (79.68%) was observed in Hybrid cultivar and the lowest (24.22%) in Ürgüp cultivar. Ion leakage in Develi cultivar was determined as 53.93%. In terms of salinity levels, the highest (64.97%) was obtained from S_5_ treatments and the lowest (42.96%) from S_1_ treatments. In terms of salinity × cultivar interaction, the greatest value was obtained from S_5_ of Hybrid cultivar and the lowest from S_1_ of Ürgüp cultivar. In previous studies on different plants, increased ion leakages were reported under abiotic stress conditions. In a study conducted on snake melon, ion leakage also increased as salinity stress increased^31^. In another study conducted in lettuce, increasing salinity also increased the ion leakage value^32^.

While salinity and cultivars had significant effects on root length (p < 0.01), salinity × cultivar interaction did not have any significant effects on root lengths. In terms of cultivars, the longest root length (9.41 cm) was obtained from Develi cultivar and the shortest (6.23 cm) from Hybrid cultivar. Root length was measured as 8.57 in Ürgüp cultivar. In terms of salinity, root lengths decreased with increasing salt doses. The longest root length (9.74 cm) was obtained from S_1_ treatment and the shortest (6.74 cm) from S_5_ treatments (Table 2). Reduction of root lengths and seedling shoots under saline conditions is a common phenomenon in many plants. Roots are the first organs to be exposed to salinity. They are in direct contact with the soil, they absorb water from the soil and transfer it to shoots^33^. Since salinity prevents the maintenance of nutrient levels necessary for plant growth through osmotic and specific ion toxicity, it also limits root development and seedling growth^34,35^. It was reported in previous studies that increasing salt doses decreased root lengths in beans^36^ and sunflowers^37^.

**Table 2:** Effect of salinity on some seedling growth parameters.

Cultivars	Treatments	Radicule (cm)	Plumule (cm)	Fresh root weight (gr)	Fresh shoot weight (gr)	Dry root weight (gr)	Dry shoot weight (gr)
Develi	S_1_	11.53^a^	3.28^ab^	0.4253^a^	0.7833^a^	0.0352^a^	0.2089
	S_2_	10.42^ab^	2.18^bcd^	0.3720^ab^	0.6423^abc^	0.0306^ab^	0.1765
	S_3_	9.12^abcd^	1.15^ cd^	0.2743^bcd^	0.5567^abcd^	0.0200^cde^	0.2178
	S_4_	8.75^abcde^	0.68^d^	0.3261^abc^	0.7331^ab^	0.0263^abc^	0.2221
	S_5_	7.22^bcde^	0.53^d^	0.1337^efg^	0.3773^d^	0.0147^defgh^	0.2172
Ürgüp	S_1_	10.43^ab^	4.97^a^	0.1837^def^	0.6040^abcd^	0.0230^cde^	0.2023
	S_2_	9.66^abc^	4.61^a^	0.2260^cde^	0.5997^abcd^	0.0212^bcd^	0.1896
	S_3_	7.11^bcde^	4.38^a^	0.1803^def^	0.5403^abcd^	0.0175^cdef^	0.2131
	S_4_	8.13^abcde^	3.32^ab^	0.1597^efg^	0.4737^ cd^	0.0159^defg^	0.2203
	S_5_	7.51^bcde^	2.96^abc^	0.1113^ fg^	0.4160^ cd^	0.0148^defgh^	0.2092
Hybrid	S_1_	7.26^bcde^	4.47^a^	0.1140^efg^	0.5837^abcd^	0.0132^efgh^	0.2223
	S_2_	6.59^cde^	3.73^ab^	0.0817^ fg^	0.4797^bcd^	0.0103^fgh^	0.2146
	S_3_	6.07^de^	3.29^ab^	0.0820^ fg^	0.4463^ cd^	0.0100^fgh^	0.2191
	S_4_	5.71^de^	3.15^abc^	0.0543^ g^	0.4327^ cd^	0.0071^gh^	0.2312
	S_5_	5.50^e^	2.99^abc^	0.0520^ g^	0.4177^ cd^	0.0063^ h^	0.2350
General means	Cultivars						
	Develi	9.41a	1.57c	0.3063a	0.6185a	0.0254a	0.2085
	Ürgüp	8.57b	4.05a	0.1722b	0.5267b	0.0185b	0.2070
	Hybrid	6.23c	3.53b	0.0768c	0.4720b	0.0094c	0.2245
	Salinity						
	S_1_	9.74a	4.24a	0.2410a	0.6570a	0.0238a	0.2112
	S_2_	8.89b	3.51a	0.2266ab	0.5739b	0.0207a	0.1936
	S_3_	7.53bc	3.14b	0.1789b	0.5144bc	0.0158bc	0.2167
	S_4_	7.43bc	2.94bc	0.1800b	0.5464b	0.0164b	0.2863
	S_5_	6.74c	2.16c	0.0990c	0.4037c	0.0120c	0.2205
Significance							
Salinity (S)		**	**	**	**	**	ns
Cultivar (C)		**	**	**	**	**	ns
S × C		ns	ns	**	*	**	ns

*ns* not significant.

*p < 0.05, **p < 0.01.

Salt doses and cultivars had significant effects on plumule length (p < 0.01), but salinity × cultivar interaction did not have any significant effects. In terms of cultivars, the lowest plumule length (1.57 cm) was obtained from Develi cultivar and the highest plumule length was obtained from Ürgüp cultivar (4.05 cm). In terms of salt doses, the highest plumule length (4.24 cm) was obtained from S_1_ treatments and the lowest from S_5_ treatments (2.16 cm). Plumule lengths decreased in all cultivars with increasing salt doses. In a study conducted on wheat, it was reported that the plumule length was affected by increasing salt doses^38^. Likewise, decreasing plumule lengths were reported with increasing salinity levels in pea^39^ and chili pepper^40^. The decrease in plumule length with increasing salinity can be explained as follows: Salinity, which is a result of osmotic pressure, causes a decrease in water absorption, thus reducing cell division and differentiation.

Salinity, cultivar and salinity × cultivar interaction had significant effects on root fresh and dry weights (p < 0.01) (Table 2). Both root fresh and dry weights decreased in all cultivars with increasing salinity levels. In terms of cultivars, the highest fresh and dry root weights were obtained from Develi cultivar (0.3063 and 0.0254 g), while the lowest values were obtained from Hybrid cultivar (0.0768 and 0.0094 g). In terms of salinity, the highest root fresh weight (0.2410 g) was obtained from control treatments and the lowest from S_5_ treatments (0.0990 g). For root dry weights, S_1_ and S_2_ treatments were placed into the same statistical group. The highest root dry weight was obtained from 0 EC treatments as 0.0238 g, while the lowest was obtained from 10 EC treatments as 0.0120 g. In terms of salinity x cultivar interaction, Develi cultivar was superior to other cultivars in terms of both fresh and dry root weight (Table 2). In terms of both root fresh and dry weights, the highest values were obtained from EC of Develi cultivar and the lowest values from 10 EC of Hybrid cultivar. In previous studies, decreasing root fresh and dry weights were reported with increasing salinity levels^3,36,41^.

Salinity and cultivar had significant effects on shoot fresh weights at p < 0.01 significance level, while salinity × cultivar interaction had significant effects at p < 0.05 significance level. In terms of cultivars, the lowest shoot fresh weight was obtained from Hybrid cultivar (0.4720 g), while the highest was obtained from Develi cultivar (0.6185 g). In terms of salinity levels, the lowest shoot fresh weight was obtained from S_5_ treatments (0.4037 g), while the highest was obtained from S_1_ treatments (0.6570 g). In terms of interaction, the highest value was obtained from S_1_ of Develi cultivar (0.7833 g) and the lowest from S_5_ of Develi cultivar (0.3773 g). Present findings comply with the results of earlier studies indicating decreasing shoot fresh weights with increasing salinity levels^30,36,42^.

Salinity, cultivar and salinity × cultivar interaction had no significant effects on shoot dry weights. In terms of cultivars, shoot dry weights varied between 0.2070 and 0.2245 g, while in terms of salinity, shoot dry weights varied between 0.1936 and 0.2863 g. Reference^43^ indicated that salt doses did not generate significant differences in shoot dry weights of peas. Reference^44^ reported that different salt concentrations did not make any significant differences on shoot dry weights of rosemary.

### Effect of salinity on mineral composition

While the effects of salinity and cultivars on root Ca contents were found to be significant at p < 0.01 significance level, they didn’t have any significant effects on shoot Ca contents. Salinity × cultivar interaction had no effect on both root and shoot Ca contents (Tables 3, 4). In terms of root Ca content of the cultivars, the highest value was obtained from Ürgüp cultivar (688.12 mg/kg) and the lowest from Hybrid cultivar (366.99 mg/kg). In terms of salinity levels, the highest value was obtained from S_1_ treatments (742.20 mg/kg) and the lowest from S_5_ treatments (397.37 mg/kg). For shoot Ca contents, the highest value was obtained from Hybrid cultivar (291.72 mg/kg) and the lowest from Develi cultivar (255.06 mg/kg). In terms of salinity levels, the greatest shoot Ca content was obtained from S_1_ treatments (309.46 mg/kg) and the lowest from S_5_ treatments (229.61 mg/kg). Calcium has significant effects on various structural and physiological processes such as cell walls, membrane structure, cell division and photomorphogenesis^45^. Seeds contain all essential plant mineral nutrients, but their availability is inhibited under stress conditions such as cold, drought and salinity^46^. In such cases, Ca^2+^ becomes important because it provides protection from stress by regulating many physiological and cellular events. However, the increase in Na ratio with the increase in NaCl dose reduces their binding by competing with Ca at the binding sites to plasma membranes^47^.

**Table 3:** Effects of different salinity levels on root mineral composition of seed pumpkin cultivars.

Cultivars	Treatments	Ca (mg/kg)	K (mg/kg)	Na (mg/kg)
Develi	S_1_	831.50	10,263.68	4006.26
	S_2_	706.32	9991.19	5815.50
	S_3_	648.28	8841.15	5344.44
	S_4_	573.16	8132.12	8428.32
	S_5_	424.82	5786.95	9394.78
Ürgüp	S_1_	883.25	15,286.37	4591.27
	S_2_	696.98	14,817.15	8137.97
	S_3_	644.72	14,796.73	9054.57
	S_4_	632.36	14,046.82	11,961.38
	S_5_	583.27	13,822.65	12,143.69
Hybrid	S_1_	511.85	9574.29	2637.58
	S_2_	449.11	8063.07	3124.52
	S_3_	341.20	7234.36	3266.10
	S_4_	348.77	5458.99d	4985.75
	S_5_	184.02	3223.25	5441.75
General means	Cultivars			
	Develi	636.82a	8603.02b	6597.86b
	Ürgüp	688.12a	14,553.95a	9177.78a
	Hybrid	366.99b	6710.79c	3891.14c
	Salinity			
	S_1_	742.20a	11,708.11a	3745.04c
	S_2_	617.47a	10,957.13a	5692.66c
	S_3_	544.74bc	10,290.75ab	5888.37bc
	S_4_	518.10bc	9212.64ab	8458.48ab
	S_5_	397.37c	7610.95b	8993.41a
Significance				
Salinity (S)		**	**	**
Cultivars (C)		**	**	**
S × C		ns	ns	ns

*ns* not significant.

*p < 0.05, **p < 0.01.

**Table 4:** Effects of different salinity levels on shoot mineral composition of seed pumpkin cultivars.

Cultivars	Treatments	Ca (mg/kg)	K (mg/kg)	Na (mg/kg)
Develi	S_1_	303.12	3579.06	348.69
	S_2_	267.55	2857.29	611.24
	S_3_	253.13	2684.35	910.82
	S_4_	242.09	2185.50	1287.23
	S_5_	209.42	2094.39	2047.29
Ürgüp	S_1_	282.51	4162.82	809.06
	S_2_	262.87	4146.77	1108.32
	S_3_	261.73	3810.30	1287.02
	S_4_	236.17	3515.32	1330.66
	S_5_	238.34	3389.01	1588.70
Hybrid	S_1_	342.74	4289.29	926.07
	S_2_	305.85	3714.84	1042.86
	S_3_	293.55	3654.58	1081.36
	S_4_	272.65	3398.11	1344.70
	S_5_	241.07	2820.10	2546.34
General means	Cultivars			
	Develi	255.06	2680.12b	1041.06
	Ürgüp	256.32	3804.85a	1224.75
	Hybrid	291.72	3575.38a	1388.27
	Salinity			
	S_1_	309.46	4010.39a	694.61c
	S_2_	278.76	3572.97ab	920.80b
	S_3_	269.47	3383.08b	1093.07b
	S_4_	250.30	3032.98b	1320.86ab
	S_5_	229.61	2767.83c	2060.78a
Significance				
Salinity (S)		ns	**	**
Cultivars (C)		ns	*	ns
S × C		ns	ns	ns

*ns* not significant.

*p < 0.05, **p < 0.01.

Salinity has significant effects on root and shoot K contents. However, salinity x cultivar interaction did not have any significant effects on root and shoot K contents. On the other hand, root K contents were significantly influenced by both cultivar and salinity (p < 0.01). Shoot K contents were significantly influenced by salinity at p < 0.01 level and cultivar at p < 0.05 level (Tables 3, 4). Both root and shoot K contents were negatively affected by increasing salinity. K contents decreased with increasing salinity levels. The highest root K content was obtained from Ürgüp cultivar (14,553.95 mg/kg), while the lowest was obtained from Hybrid cultivar (6710.79 mg/kg). For shoot K contents, the highest was obtained from Ürgüp cultivar (3804.85 mg/kg), while the lowest was obtained from Develi cultivar (2680 mg/kg). In terms of salinity levels, the highest root and shoot K contents were obtained from control treatments (117,081.11 mg/kg and 4010.39 mg/kg), while the lowest values were obtained from 10 EC treatments (7610.95 mg/kg and 2767.83 mg/kg). K participates into many cellular functions such as activation of enzymatic reactions, load balancing and osmoregulation^48^. Therefore, K plays an important role in salinity stress tolerance of the plants. Salinity may result in plant nutritional disorders such as suppression of K absorption^49^. Decreasing K contents were also reported in sunflowers with increasing salinity levels^50^. Salinity stress decreases total K accumulation in plants and has negative effects on plant growth and development^51^. Reference^52^ represented that increasing NaCl levels caused an increase in K leakage from the seeds.

Salinity and cultivar had significant effects on root Na contents at p < 0.01 significance level, but only salinity had significant effects on shoot Na contents (p < 0.01). The salinity × cultivar interaction did not have any significant effects on both root and shoot Na contents (Tables 3, 4). Root and shoot Na contents increased with increasing salinity levels. The highest Na content was obtained from Ürgüp cultivar (9177.78 mg/kg) and the lowest from Hybrid cultivar (3891.14 mg/kg). For shoot Na contents, the lowest (1041.06 mg/kg) value was obtained from Develi cultivar, while the highest value (1388.27 mg/kg) was obtained from Hybrid cultivar. In terms of salinity levels, the lowest root and shoot Na contents were obtained from S_1_ treatments (3745.04 and 694.61 mg/kg) and the highest values were obtained from S_5_ treatments (8993.41 and 2060.78 mg/kg). Plant roots had greater Na contents than the shoots. High root Na levels can maintain the normal osmotic potential and prevent the transport of this ion, thus preventing the accumulation of Na in the other organs^53^. Increasing root and shoot Na contents were reported in previous studies with increasing salinity levels^50,54,55^. Under high salinity levels, Nam ay reduce N-compounds and thus slow down transport rate of essential ions, which ultimately inhibiting plant growth and biomass accumulation^56,57^.

**Figure 1:**
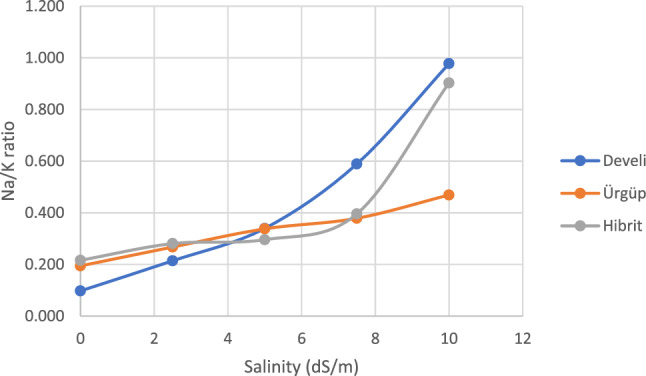
Seedling Na:K ratio of seed pumpkin cultivars.

It is important to determine the Na, K and Na:K ratio in order to understand the salinity tolerance mechanisms^58^. The change in Na:K ratio is presented in Fig. 1. In our study, it was observed that there was a significant difference between varieties under salinity stress. The Na:K ratios increased with increasing NaCl doses. While this increase was lower in Ürgüp cultivar, it was observed to be almost the same in Develi and Hybrid cultivars.

The increase in Na ion content and decrease in K ion uptake cause ionic imbalance, inhibition of K transport process of Na in vascular tissues and Na-induced K flux from the roots as there is direct competition between these two ions. Reference^59^ explained that ion antagonism occurred when nutritional imbalance was encountered due to salinity. Na increased proportionally with different salinity levels in both root and shoot, but the rate of increase was higher in root. Therefore, ionic ratios are important keys for determining relative toxicities that can provide relative biological process rates under certain ionic antagonisms. In fact, in many species, it is vital to maintain a high K/Na rather than a low Na concentration. Parallel results were obtained in studies conducted on the other plants^50,60^.

### Effect of salinity on antioxidant capacity, prolin, total phenolic and DPPH

The effects of different salinity levels on antioxidant capacity, proline, total phenolic and DPPH content in pumpkin seed varieties are given in Table 5.

**Table 5:** Effects of different salinity levels on antioxidant capacity, prolin, total pheneolic, DPPH.

Cultivars	Treatments	Prolin (mg/g)	Antioxidant capacity (mg AAE/gr)	Total phenolic (mg GAE/g)	DPPH (%)
Develi	S_1_	1.12abc	15.33a	6.02ab	26.48b
	S_2_	1.28ab	9.67b	7.99a	38.65a
	S_3_	1.35a	7.68bcd	4.99bc	21.04bc
	S_4_	1.20abc	6.01cde	3.94bcde	14.30de
	S_5_	1.13abc	4.08efg	2.71cdef	4.73gh
Ürgüp	S_1_	0.73bc	7.89bc	4.90bc	19.98 cd
	S_2_	0.83abc	7.73bcd	4.52bcd	19.74 cd
	S_3_	1.25ab	5.26def	3.40bcdef	9.88efg
	S_4_	1.38a	2.60 g	1.70ef	5.32fgh
	S_5_	1.39a	1.88 g	1.02f.	1.18 h
Hybrid	S_1_	0.68c	14.72a	8.14a	41.96a
	S_2_	0.90abc	7.43bcd	5.27bc	21.87bc
	S_3_	1.18abc	5.28cdef	3.40bcdef	11.11e
	S_4_	1.32a	3.92efg	3.16cdef	10.76ef
	S_5_	1.39a	2.85 fg	2.01def	4.97gh
General means	Cultivars				
	Develi	1.22	8.55a	5.13a	21.04a
	Ürgüp	1.12	5.07c	3.11b	11.22c
	Hybrid	1.09	6.84b	4.40a	18.13b
	Salinity				
	S_1_	0.84c	12.65a	6.35a	29.47a
	S_2_	1.00bc	8.28b	5.93a	26.75b
	S_3_	1.26ab	6.07c	3.93b	14.01c
	S_4_	1.30a	4.18d	2.93bc	10.13d
	S_5_	1.30a	2.93e	1.91c	3.63d
Significance					
Salinity (S)		**	**	**	**
Cultivars (C)		ns	**	**	**
S × C		*	**	*	**

*ns* not significant.

*p < 0.05, **p < 0.01.

Different salinity levels had statistically significant effects on the proline content (Table 5). While the difference between the cultivars was nonsignificant, the salinity × cultivar interaction was significant at p < 0.05 level. Proline content increased with increasing salt doses. In Develi cultivar, a decrease occurred after the salinity dose of 5 dS/m. The highest proline content was obtained from the S_4_ and S_5_ treatment, while the lowest was from the S_1_ treatment.

Proline is one of the common osmolytes that maintains fluid balance in plants and is up-regulated in stress situations and provides protection against damage^61^. Salt stress disrupts the composition of cellular ions, causing ion toxicity and osmotic stress^62^. To cope with osmotic stress and consequent damage under salt stress, plants begin to produce and accumulate non-enzymatic antioxidant solutes such as proline and ascorbate as well as other enzymatic antioxidants^62,63^. In previous studies, it has been reported that there is an increase in the amount of proline in parallel with the increase in the salinity level^64–66^.

While cultivar and salinity were effective on total phenolic content at p < 0.01 significance level, salinity × cultivar interaction was effective at p < 0.05 significance level. The highest total phenolic content among the cultivars was found in Develi (5.13 mg GAE/g), there was no statistical difference between Hybrid cultivar (4.40 mg GAE/g). The total phenolic content of Urgüp cultivar was found to be 3.11 mg GAE/mg. In terms of salinity, the highest total phenolic content was taken from S_1_ and S_2_ treatments, while the lowest was from S_5_. According to the results of the study, increasing salinity levels caused significant decreases in total phenolic content. While the total phenolic content of Develi cultivar increased up to 2.5 dS/m salinity level, significant decreases occurred after this level. In Urgüp and Hybrid varieties, the total phenolic content decreased in all treatments after control treatment. In terms of interaction, the highest total phenolic content was obtained from Hybrid S_1_ and Develi S_2_ subjects, while the lowest was from Urgüp S_5_.

It is well known that abiotic stresses, including salinity, cause oxidative damage mainly by generating excess ROS (reactive oxygen species) that can attack lipids, proteins, DNA and carbohydrates. ROS consistent of both non-radical (O_2_ ve H_2_O_2_) and free radical forms (OH, O_2_^−^ , RO ve HO_2_)^67^. To scavenge ROS, antioxidants such as phenolic compounds are produced by plants, and thus the biosynthesis of such compounds is often stimulated in plants exposed to salt^68^. Findings parallel to the results of the study have also been reported in studies on different plants^69–71^.

Salinity, cultivar and salinity × cultivar interaction had significant effects on DPPH (Table 5). Among the cultivars, the highest DPPH content was obtained in Develi (21.04%) and the lowest in Ürgüp (11.22%). Decreases in DPPH content occurred with increasing salinity level in pumkin seed. The highest DPPH content was obtained from the control treatment (29.47%), while the lowest was obtained from the salinity level of 10dS/m. There was an 87.7% reduction in DPPH content compared to the control treatment. Compared to the control treatment, a reduction of 47.9% occurred at the salinity level of 2.5 dS/m in the hybrid cultivar. Reference^72^ in cotton, Ref.^73^ in coriander reported that DPPH content decreased with increasing salinity level in their studies. According to the results of the study, it was determined that the total phenolic content and DPPH results were similar. These antioxidant capacities may be directly related to the amounts of phenolic compounds due to their free radical scavenging capacity^74^.

The effect of different salinity applications on antioxidant capacity content in pumpkin seed, the effect of cultivar, salinity and salinity × cultivar interaction created a difference at p < 0.01 significance level. Among the cultivars, Develi was the variety with high antioxidant capacity, followed by Hybrid and Urgüp, respectively. (Table 5). With increasing salt doses, the content of antioxidant capacity decreased. The highest is taken from the S_1_ subject, while the lowest is from the S_5_ treatments. In terms of interaction, the highest S_1_ treatment (15.33 and 14.72 mg AAE/gr) was obtained from Develi and Hybrid cultivars, while the lowest was obtained from Urgüp S_5_ treatment (1.88 mg AAE/gr).

Phenolic compounds show antioxidant activity by inactivating lipid free radicals or preventing the decomposition of hydroperoxides into free radicals^75^. The degree of cellular oxidative damage in plants exposed to abiotic stress is controlled by the plants' capacity to produce antioxidant agents. However, its accumulation under salinity conditions varies considerably among plants. According to Ref.^76^ *Salvia mirzayanii*, Ref.^77^ *Carthamus tinctorius* L. increased with increasing salinity, Ref.^78^ reported the opposite results in their study on lettuce.

**Figure 2:**
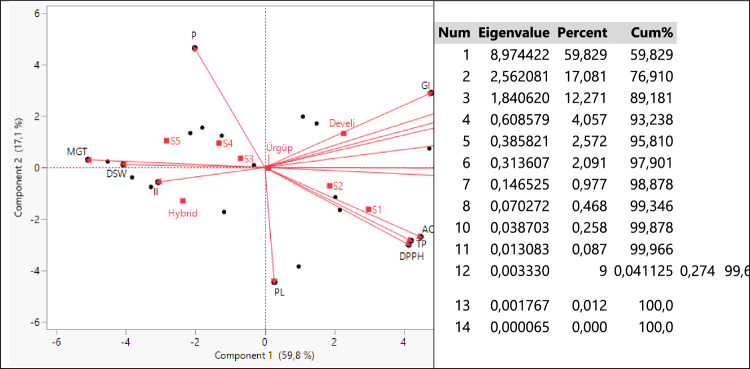
Biplot analysis on genotypes, applications and parameters studied and their values.

Axes, eigenvalues, variance and total variance values obtained with the biplot analysis output of the parameters examined at different salinity levels of three pumpkin seed genotypes are given in Fig. 2.

When Fig. 2 is examined, 3 principal component axes with eigenvalues higher than 1 have formed a total of 14 principal component axes that are independent of each other. PC1 and PC2 defined 76.91% of the total variation, while their eigenvalues were recorded as 8.97 and 2.56, respectively. These outputs show that the biplot analysis can be interpreted successfully^79,80^. When the lengths of the axes, their angles with each other and the regions where they are clustered are examined, the GI, FRW, GP, DRW and RL parameters are; AC, TP and DPPH parameters; MGT, DSW and II parameters were highly correlated with each other. The Develi genotype was a pioneer especially in GI, FRW and GP parameters. Hybrid genotype was separated from other genotypes in terms of II parameter and had the highest value. Since Urgup genotype is located close to the origin, it has been a genotype with average values in terms of all applications and parameters examined.

## Scatter plot matrix for overview of correlations and fit lines

**Figure 3:**
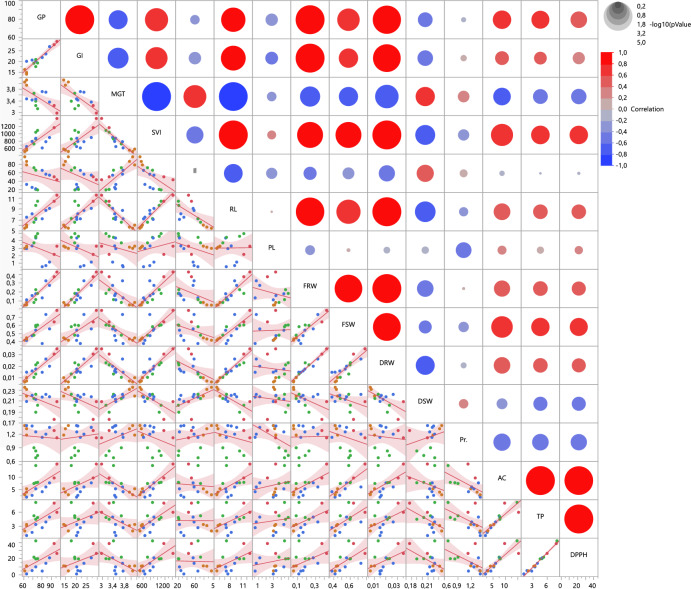
Scatter plot and matrix for overview of correlations and fit lines. *GP* germination percentage, *GI* germination index, *MGT* mean germination time, *SVI* seedling vigor index, *Il* ıon leakage, *RL* radicule lenght, *PL* plumule lenght, *FRW* fresh root weight, *FSW* fresh shoot weight, *DRW* dry root weight, *DSW* dry shoot weight, *Pr* prolin, *AC* antioxidant capacity, *TP* total phenolic.

The correlation relationship between the data obtained as a result of the study is shown in Fig. 3.

While red circles show the positive relationship between the parameters examined, blue circles show the negative relationship. In addition, the size of the diameter of the circle indicates the degree of the relationship. Likewise, the distribution of colored genotypes obtained from the correlation server around the fitline lines obtained as a result of the analysis output can be seen in Fig. 3. Among the traits examined by seedling vigor index (SVI), which is an important parameter in seed germination, a positive correlation was observed between GP (r^2^ = 0.774), GI (r^2^ = 0.745), RL (r^2^ = 0.929), FRW (r^2^ = 0.837), FSW (r^2^ = 0.836), DRW (r^2^ = 0.894), AC (r^2^ = 0.747), TP (r^2^ = 0.640) and DPPH (r^2^ = 0.635). It was determined that there were negative correlations between SVI and MGT (r^2^ = − 0.902), II (r^2^ = − 0.588), DSW (r^2^ = − 0.682) and PR (r^2^ = -0.344). While all germination parameters except MGT had a positive correlation with growth parameters, a positive correlation was found only between MGT and DWS (r^2^ = 0.661). While there was only a weak positive correlation between MGT and biochemical properties with Pr (r^2^ = 0.361), negative correlations were found between AC (r^2^ = − 0.605), TP (r^2^ = − 0.499) and DPPH (r^2^ = − 0.521).

## Conclusion

This study was carried out to determine the responses of pumpkin seed varieties to different salinity stress. In the study, GP, MGT, ion leakage and SVI were affected by both salinity and variety. The increase in salinity level caused an average decrease of 16.1% in GP, a 15.5% increase in MGT, a 33.9% increase in ion leakage and a 46.9% decrease in SVI. The GI value was affected only by the variety and the highest value was obtained from the Develi variety. When the growth parameters were examined, radicule, plumule, fresh root and shoot weight, dry root weight were affected by both variety and salinity. The increase in salinity had negative effects on growth parameters. Ca, K and Na were examined as mineral composition, and both salinity and variety created a statistical difference. While the increase in salinity caused a decrease in Ca and K content, it caused an increase in Na content. When the Na/K ratio was examined, it was seen that Develi variety differed from other varieties. In this study, proline, antioxidant capacity, total phenolic and DPPH content were examined as biochemical content. As a result of the experiment, while proline level increased in parallel with the increase in salinity, other parameters decreased with the increase in salinity. It was seen that especially in semi-arid climate regions, Develi cultivar may be advantageous as compared to other cultivars in saline lands.

## Data Availability

The datasets used and/or analyzed during the current study available from the corresponding author on reasonable request.
